# Early changes in apoptosis and proliferation following primary chemotherapy for breast cancer

**DOI:** 10.1038/sj.bjc.6601173

**Published:** 2003-09-09

**Authors:** C D Archer, M Parton, I E Smith, P A Ellis, J Salter, S Ashley, G Gui, N Sacks, S R Ebbs, W Allum, N Nasiri, M Dowsett

**Affiliations:** 1 Breast Unit, Royal Marsden NHS Trust, Fulham Road, London SW3 6JJ, UK; 2 Academic Department of Biochemistry, Royal Marsden NHS Trust, Fulham Road, London SW3 6JJ, UK; 3 Academic Department of Computing, Royal Marsden NHS Trust, Fulham Road, London SW3 6JJ, UK; 4 Mayday University Hospital, London Road, Croydon, Surrey CR7 7YE, UK; 5 Academic Department of Pathology, Royal Marsden NHS Trust, Fulham Road, London SW3 6JJ, UK

**Keywords:** breast cancer, apoptosis, proliferation, chemotherapy

## Abstract

Patients undergoing primary chemotherapy for invasive breast cancer consented to a core biopsy of the invasive breast primary pre- and 24 h postchemotherapy. The resulting tissue was analysed for apoptosis, Ki67, ER and HER-2 using immunohistochemical techniques. These data were then used to evaluate the relationship between these biological markers and response to chemotherapy and overall survival. Response rate to chemotherapy in this group was 86%, 16 patients (25%) achieved a clinical complete response and 41 (63%) a partial response. Prechemotherapy there was a significant correlation between Ki67 and apoptotic index (AI), *r*=0.6, (*P*<0.001). A significant rise in AI (*P*<0.001), and fall in Ki67 (*P*=0.002) was seen 24 h following chemotherapy. No relationship was seen between pretreatment AI and clinical response, but higher Ki67 and growth index (Ki67/AI ratio, GI) did correlate with clinical response (both *r*=0.31, *P*<0.025). No correlation was seen between the change in AI or Ki67 at 24 h and clinical response or survival. Significant changes in apoptosis and proliferation can be demonstrated 24 h following chemotherapy, but these changes do not relate to clinical response or outcome in this study. Pretreatment proliferation and GI are however predictive of response to chemotherapy in breast cancer.

The use of primary or preoperative chemotherapy emerged as a result of the good response rates seen with the treatment of locally advanced breast cancers with chemotherapy (Swain *et al*, 1987; Hortobagyi *et al*, 1988). In large but operable tumours, primary chemotherapy has also shown high response rates, allowing more conservative surgery. This reduces the need for mastectomy, without affecting the local recurrence rate (Powles *et al*, 1995; Smith *et al*, 1995; Fisher *et al*, 1997), and without compromising survival (Fisher *et al*, 1998).

Another advantage of primary chemotherapy is that it provides an *in vivo* model by which to study the effects of chemotherapy on the primary tumour (Forrest *et al*, 1986). Adjuvant chemotherapy has improved the outcome for women with breast cancer, through large clinical trials (EBCTCG, 1998a). These trials, however, require many years of follow-up and very large numbers of patients. New approaches are needed to allow more rapid assessment of the large number of new agents presently under development. Clinical response to primary chemotherapy is associated with improved survival, (Cameron *et al*, 1997; Bonadonna *et al*, 1998; Fisher *et al*, 1998), but this is an insensitive surrogate since the majority of patients achieve a response. Biological markers, which are predictive of response, might prove to be more sensitive short-term surrogates of long-term outcome. If this were the case, the use of these markers would substantially increase the speed of drug development, and also tailor chemotherapy more effectively for the individual patient.

Studies in primary chemoendocrine and endocrine therapy have shown that early changes in proliferation, 14–21 days after starting therapy, are significantly associated with clinical response. (Dowsett *et al*, 1999). An increase in apoptosis following cytotoxic chemotherapy occurs much earlier. In murine mammary tumours, apoptosis has been demonstrated within 24–36 h of cytotoxic drug administration (Meyn *et al*, 1994,^1995^), and in our earlier preliminary clinical studies significantly increased apoptosis was seen 24 h after starting chemotherapy (Ellis *et al*, 1997). A small study has indicated that an early increase in apoptosis as measured in fine-needle aspirates (FNA) by flow cytometry was significantly associated with clinical response in breast cancer, but this methodology was noted to be complicated by problems of precision (Chang *et al*, 1999). This study used core biopsy material to evaluate the changes in apoptosis and proliferation 24 h after chemotherapy, and assessed the relationship of those changes with clinical response and survival.

## MATERIALS AND METHODS

### Patient population

The study period was between February 1995 and March 2001. Patients presenting to the Royal Marsden Hospital with nonmetastatic primary breast cancer of 3 cm or greater and to be treated with primary chemotherapy were eligible for entry into this study. The diagnosis of invasive breast cancer was made on 14-gauge core biopsy in all patients prior to primary chemotherapy. Following informed consent, patients entering the study had a further core biopsy 24 h after the start of the first course of chemotherapy, and the material in both biopsies was analysed.

### Treatment

Chemotherapy was anthracycline-based, except two patients who received mitoxantrone. In all, 34 patients received doxorubicin-based, and 30 patients epirubicin-based combination chemotherapy. A total of 38 patients were entered into trials of primary chemotherapy during the study period (Smith *et al*, 1995,^2000^; Eisen *et al*, 1998), others were treated with standard combination chemotherapy. Tamoxifen was also prescribed to 49 (74%) patients, 27 of whom started tamoxifen during the chemotherapy (although after the 24 h biopsy), and the remainder following completion of chemotherapy. Chemotherapy was continued for six cycles or until progression, when patients were considered for surgery, which was usually lumpectomy or mastectomy with axillary clearance.

### Evaluation of tumour response

Response was assessed by bidimensional tumour measurements before each cycle of chemotherapy. Response was defined as per WHO criteria (Miller *et al*, 1981). Pathological evaluation of the tumour postsurgery identified those who had no residual invasive or *in situ* disease pathological complete response (pCR).

### Laboratory methods

Core biopsies were taken after local anaesthetic infiltration of the skin using a 14-gauge needle on a spring-loaded device. The material obtained was formalin-fixed and paraffin-embedded. Sections (3 *μ*m) were cut onto charged slides and left at 37°C overnight. These were examined by a Consultant histopathologist (NN), for histological classification (NCGFBS, 1995) and grading where possible (Bloom and Richardson, 1957). Immunohistochemistry for Ki67, ER and HER-2 was performed using a standard avidin–biotin complex technique according to the following methodology, and apoptotic index (AI) assessed using Terminal deoxynucleotidyltransferase-mediated d-UTP nick end labelling (TUNEL) and *in situ* end-labelling (ISEL) techniques.

#### Ki67

Sections were dewaxed, hydrated and taken to water and treated with hydrogen peroxidase to neutralise endogenous peroxidases. After antigen retrieval by microwaving in citrate buffer for 5 min at full power (750 W microwave), normal rabbit serum at a dilution of 1:5 was applied. MIB1 primary antibody (The Binding Site Ltd, UK) was used at a dilution of 1:50, and incubated for an hour at room temperature. All dilutions and washes were with phosphate-buffered saline (PBS). Rabbit anti-mouse serum was applied followed by avidin–biotin complex (ABC) (Dako, Denmark), which was developed by diaminobenzene (DAB) (Sigma, USA), and counterstained with haematoxylin.

#### HER-2

The sections were treated in a similar fashion as with the MIB1 antibody, except that antigen retrieval was not required. The primary antibody, ICR 12 (Gusterson *et al*, 1992) was used at a dilution of 1:800, incubated for an hour at room temperature.

*Oestrogen receptor* (ER) The same staining procedure as for MIB1, with microwave antigen retrieval. The primary antibody used was ID5 (Dako) incubated at a dilution of 1:100 for 2 h at room temperature, according to previously validated conditions (Saccani Jotti *et al*, 1994).

*Apoptotic index In situ* end labelling (ISEL) uses biotin-16-dUTP (Boehringer Mannheim) plus the Klenow fragment of *Escherichia coli* DNA polymerase 1 (Pharmacia) (Wijsman *et al*, 1993). Terminal deoxynucleotidyltransferase-mediated d-UTP nick end labelling (TUNEL) technique is modified from Gavrieli *et al* (1992). Both these techniques have been used by our laboratory and have shown equivalent results in breast cancer sections (Mainwaring *et al*, 1998).

### Scoring

All sections were coded and scored blind to time of biopsy, treatment and clinical outcome by PAE, CDA and MP. Sections were examined under a standard light microscope using a × 40 objective and a 10 × 10 eye piece incorporating a graticule. Invasive breast cancer cells only were counted. Ki67 score was defined as a percentage of total number of tumour cells with nuclear staining to the MIB1 antibody over 10 high power fields (× 40). A tumour was defined as HER-2 positive by the presence of the characteristic membrane staining in over 10% of the tumour cells. The AI was a percentage score of apoptotic cells from a total of 3000 malignant cells. Unstained apoptotic cells were included if they showed classical morphological features of apoptosis, cytoplasmic condensation and chromatin clumping (Wyllie *et al*, 1980). Apoptotic bodies not obviously associated with an apoptotic cell were included in the count only when present as clumps. Oestrogen receptor was assessed either with a Histo-score (*H*-score) or using a Quickscore that has been previously validated against *H*-score in breast cancer sections. Oestrogen receptor positive was defined as an *H*-score of >20 (Detre *et al*, 1995).

### Statistical analysis

Nonparametric statistics were used. The changes in AI and proliferation during the first 24 h of chemotherapy were analysed using the Wilcoxon signed-rank test. The relationship at baseline between AI and MIB1 scores was assessed using the Spearman rank correlation, as was the association between biological markers and response. Kaplan–Meier curves were used for survival analysis. No formal powering of the study was conducted, but with 66 patients it would be possible to detect a correlation of 0.40 or greater with 90% power.

### Ethical

This study was approved by the Research and Ethics Committee of the Royal Marsden NHS Trust. All patients gave written informed consent.

## RESULTS

### Clinical results

A total of 108 patients consented to study entry and had a repeat core biopsy 24 h after starting chemotherapy. In all, 66 patients had sufficient invasive tissue in both biopsies to be used for analysis of the biological markers, although seven of these did not have enough material to be tested for all parameters. The characteristics of the study population are given in Table 1

**Table 1:** Demographics and patient characteristics

**Characteristic**	**No. of patients (*n*=66)**
Age (years)	
Median	48 (32–63)
Menopausal status	
Premenopausal	39 (59%)
Perimenopausal	3 (5%)
Postmenopausal	24 (36%)
Tumour stage	
T-2	29 (44%)
T-3	26 (39%)
T-4 operable	1 (2%)
T-4 inoperable	10 (15%)
Tumour size (cm)	
Median	5.7 (3–20)
Histology	
Ductal	56 (84%)
Lobular	8 (12%)
Ductal+lobular	1 (2%)
Mucinous	1 (2%)
Grade	
Grade1	0 (0%)
Grade 2	29 (44%)
Grade 3	34 (51.5%)
Unknown	3 (4.5%)
ER status	
Positive	43 (65%)
Negative	23 (35%)
HER-2 status	
Positive	18 (27%)
Negative	48 (73%)

.

Details of clinical and surgical response to treatment are shown in Table 2

**Table 2:** Overall response to treatment and outcome

	**No. of patients**
Response to primary chemotherapy	*n*=65
Complete response	16 (24%)
Partial response	41 (62%)
Overall response rate	57 (86%)
No change	6 (9%)
Progressive disease	2 (3%)
Surgery	*n*=66
Conservative	36 (55%)
Mastectomy	17 (26%)
No surgery	13 (19%)
Pathological status at surgery	*n*=66
Residual invasive disease	46 (70%)
Complete pathological response	5 (7.5%)
DCIS only	3 (4.5%)
Overall progression-free survival	*n*=66
1 year	87%
2 years	74%
3 years	65%
Overall survival	*n*=66
1 year	93%
2 years	83%
3 years	77%

DCIS=ductal carcinoma *in situ*.

. In all, 57 patients (86%) responded to treatment, 16 (25%) achieving a clinical complete remission (cCR) and 41 (63%) a partial response (PR). One patient was not evaluated for response due to death early on treatment. Following chemotherapy, 12 patients did not have surgical treatment: one refused, two remained inoperable and nine patients achieved a cCR and had radiotherapy alone, as part of a separate research study. As expected, this overall pCR rate of 7.5% is lower than the 16% seen in the major primary chemotherapy trial recruiting in our institution during the study period (Smith *et al*, 2000). This was clearly influenced by the nine patients (18%) with a cCR who did not have any surgery following chemotherapy. Median follow-up was 36 months (1–74) and there were 21 relapses and 15 deaths. Progression-free and overall survival figures are detailed in Table 2.

### Biological results

#### Apoptosis and proliferation

**Figure 1:**
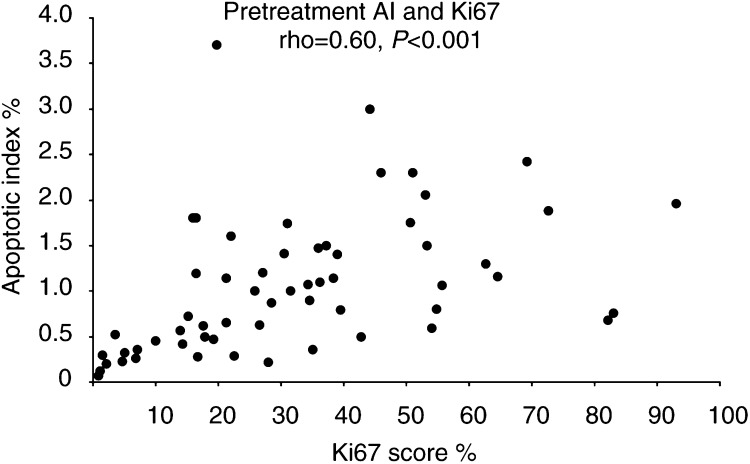
Correlation of pretreatment Ki67 and AI scores. Each data point represents the scores for an individual tumour (rho=0.60, *P*<0.001).

Prior to treatment there was a highly significant positive correlation between proliferation, as measured by Ki67 and AI samples (rho=0.60, *P*<0.001), Figure 1

**Figure 2:**
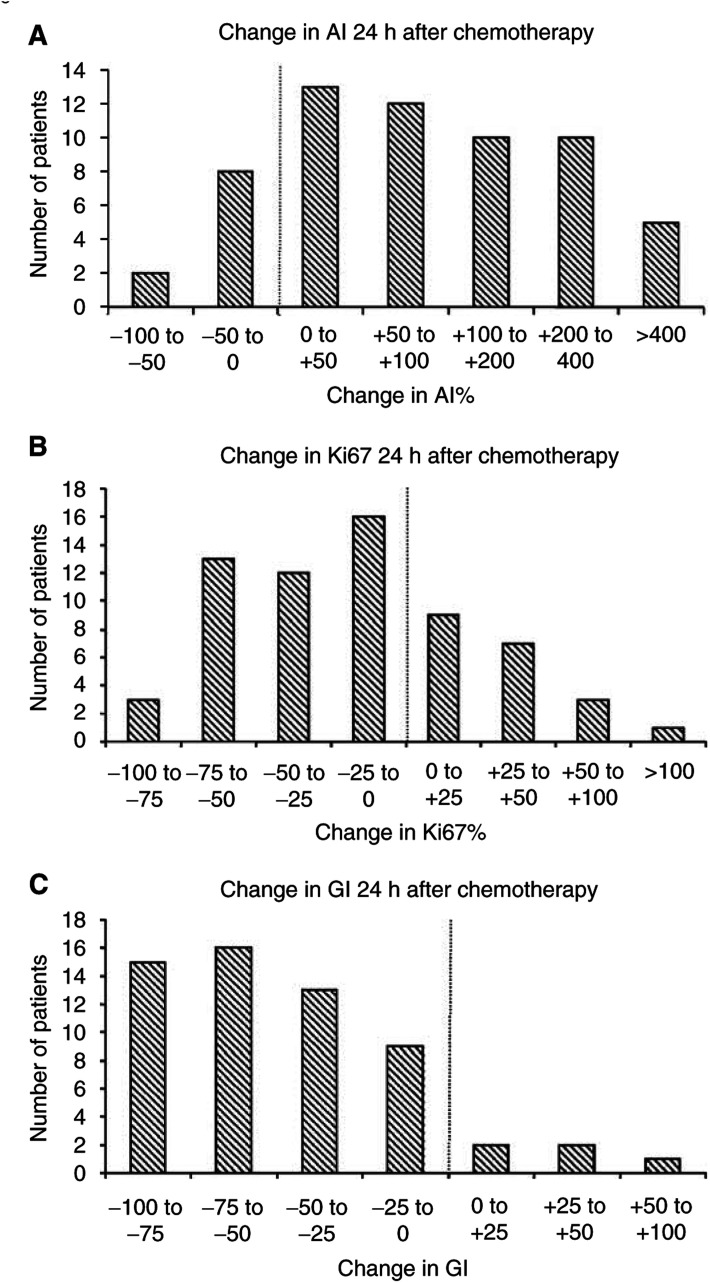
The percentage change in Al (**A**), Ki67 (**B**) and GI (**C**) in core biopsies 24 h after chemotherapy (*n*=60). The scores have been divided to show a fall to the left of the line and a rise to the right, (**A**) AI, *P*<0.001, (**B**) Ki67, *P*=0.002, (**C**) GI *P*<0.001).

. Tumour grade was also significantly correlated with both apoptosis and proliferation pretreatment, *P*<0.001. Median AI prechemotherapy was 0.9 (interquartile range (IQ), 0.5–1.5) and at 24 h postchemotherapy this had increased significantly to 1.60 (IQ 1.0–2.9, *P*<0.001), Figure 2A

. The median Ki67 score pre-chemotherapy was 28.5 (IQ 16.1–43.9) and 24 h following chemotherapy this had fallen significantly to 17.6 (IQ 8.7–26.8, *P*=0.002), Figure 2B.

In addition to assessing the impact of chemotherapy on Ki67 and AI individually, the effect on the Ki67/AI ratio, a parameter which we have described as a growth index (GI) (Harper-Wynne *et al*, 2002) was assessed. There was a significant fall in GI at 24 h, from 28.5 (IQ 19.9–43.5) to 11.5 (IQ 6.0–25.2, *P*<0.001), Figure 2C. It was notable that while Ki67 increased in 20 patients and AI decreased in 10 patients, only five patients showed an increase in GI.

The changes in AI and Ki67 are shown according to HER-2 and ER status in Table 3

**Table 3:** Percentage change from pretreatment levels in AI, Ki67 and GI after 24 h of chemotherapy

	**AI**	**Ki67**	**GI**
All			
N	60	64	58
Median	+81.1	−17.0	−53.4
IQ range	+12.7 to +205.65	−50.0 to +8.5	−76.5 to −27.4
ER positive			
N	38	**43**	38
Median	+82.5	**−26.1**	−62.6
IQ range	+4.6 to +237.5	**−55.7 to −1.11**	−80.2 to −29.4
ER negative			
N	22	**21**	20
Median	+81.0	**−3.2^a^**	−31.3
IQ range	+29.8 to +167.5	**−26.8 to +33.0**	−60.6 to −21.4
HER-2 positive			
N	15	18	15
Median	+33.1	−12.9	−37.4
IQ range	+10.8 to +154.6	−37.3 to +14.0	−77.3 to −4.7
HER-2 negative			
N	45	46	43
Median	+100.0	−19.4	−54.0
IQ range	+14.85 to +221.4	−54.6 to +9.3	−76.3 to −28.9

a Change in Ki67 between ER-positive and ER-negative tumours, *P*=0.006.

. There were no significance differences in the changes in apoptosis and proliferation seen with chemotherapy according to HER-2 status, but those patients who were ER-positive had a greater proportional fall in proliferation postchemotherapy compared with ER-negative patients (*P*=0.006).

### Relationship of biological indices with clinical response

#### Pretreatment indices

There was no relationship between pretreatment AI and overall clinical response, *r*=−0.04, but higher levels of proliferation pretreatment correlated with increased clinical response, *r*=−0.31, *P*<0.025. Tumour grade was not correlated with overall clinical response, but grade 3 tumours had a greater rate of cCR rate than grade 2, *P*=0.007. A significant relationship was also seen between the GI pretreatment and the clinical response to treatment, *r*=0.31, *P*<0.025. A significant correlation between both pretreatment proliferation and GI (*P*<0.025) with clinical response was also seen in the subgroup of patients treated with chemotherapy alone. These were defined as no concomitant tamoxifen or ER-negative patients who received tamoxifen, where the effects would have been expected to be negligible. No correlation was seen with pretreatment AI and clinical response in this subgroup.

#### Changes at 24 h

**Figure 3:**
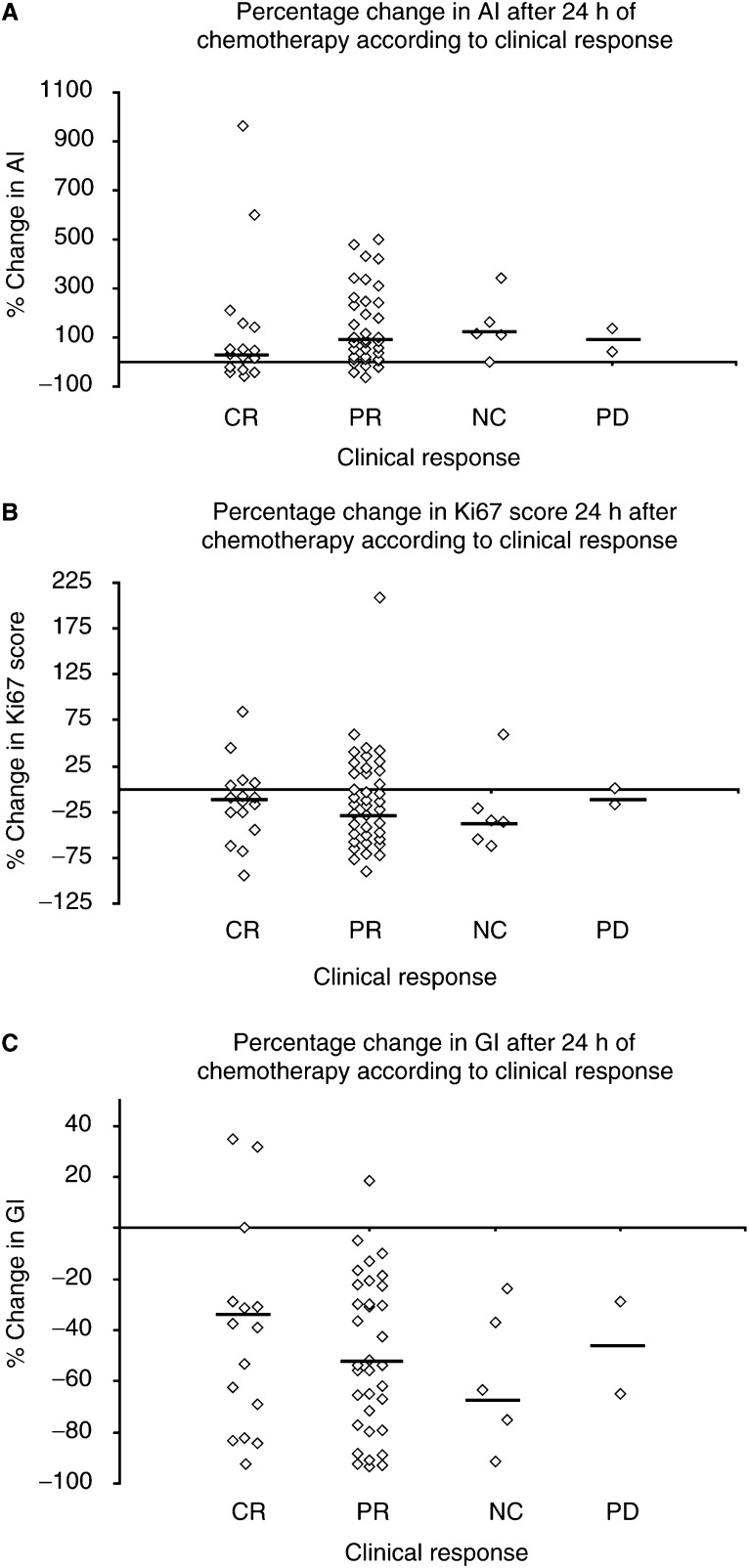
Scattergram illustrating the relationship between change in AI (**A**), Ki67 (**B**), GI (**C**) and clinical response. Each dot is a value for a tumour and the horizontal line is the median value. PD, progressive disease; NC, no change

There was no significant relationship seen between clinical response and the biological changes 24 h after chemotherapy for either AI, Ki67 or GI, Figure 3

. This applied to either the whole group or that of the chemotherapy alone group.

### Relationship of biological indices with progression-free and overall survival

**Figure 4:**
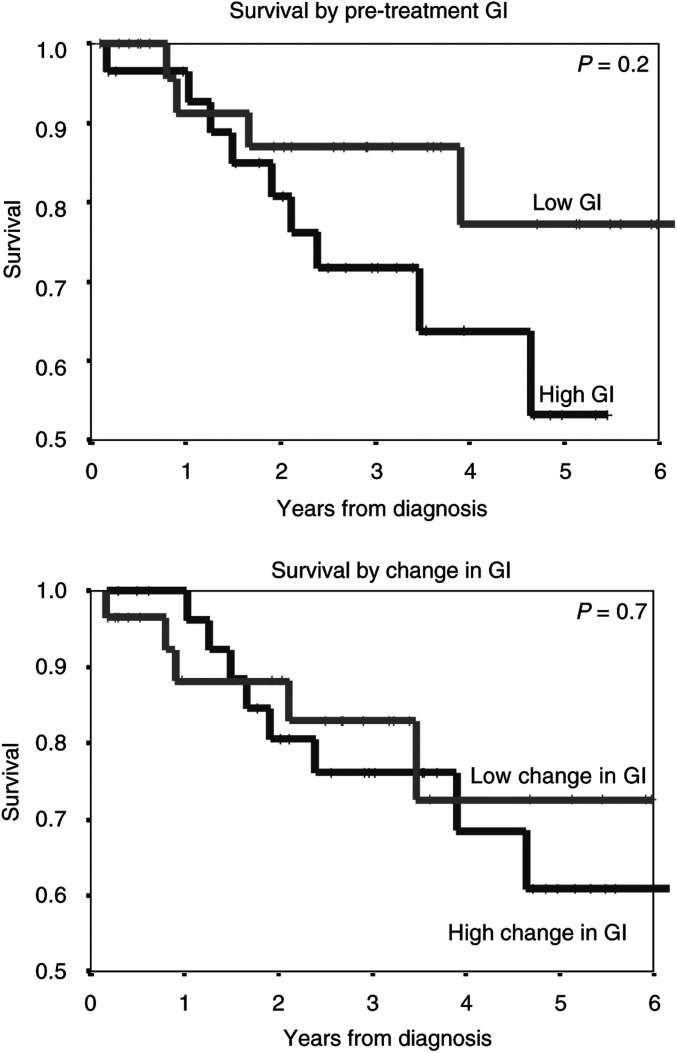
Overall survival curves for patients according to pretreatment tumour growth indices (GI) (low GI<28.5 (median value) and high GI >28.5), and change in GI 24 h after chemotherapy (low change in GI <53.4 (median value), and high GI >53.4).

On univariate analysis, no relationship was seen between pretreatment AI, Ki67 or GI (assessed as high or low values, above or below the median) with progression-free or overall survival. Similarly, there was no significant relationship between the changes in these parameters seen at 24 h after chemotherapy with progression-free or overall survival. Although those patients with a lower pretreatment GI appeared to have an improved overall survival this was not significant, *P*=0.2, Figure 4

.

## DISCUSSION

In this study, we have demonstrated a significant relationship between AI and Ki67 in breast tumours prior to the initiation of therapy. This is consistent with our previous work (Ellis *et al*, 1998) and that of others (Lipponen *et al*, 1994; van Slooten *et al*, 1998), indicating that tumours with a high rate of apoptosis are also rapidly proliferating. Apoptotic index and Ki67 also correlated positively with high pathological grade. Apoptotic index has been previously shown to be associated with p53 overexpression, but inversely with bcl-2 and ER (Lipponen *et al*, 1994; Hori *et al*, 1997; Rochaix *et al*, 1999). A high proliferation rate in breast tumours is associated with a poorer prognosis (Meyer and Lee, 1980; Silvestrini *et al*, 1985) as is p53 expression (Silvestrini *et al*, 1993), whereas ER overexpression is associated with a more favourable prognosis (EBCTCG, 1998b). One might also expect AI to be associated with a poorer outcome, but AI has not been found to have independent prognostic value (Lipponen *et al*, 1994).

Pretreatment levels of proliferation positively correlated with clinical response. This is consistent with other studies, either using the MIB1 antibody or S-phase fraction (Bonetti *et al*, 1996; Chevillard *et al*, 1996; MacGrogan *et al*, 1996). Ki67 is an antigen that binds to a protein expresssed in late G1-, S-, G2-, and M-phase of the cell cycle, and although most cytotoxic drugs have maximum effect in S-phase, some also act in other phases of the cell cycle, and therefore this finding would be compatable with the biology (Boyer and Tannock, 1998). It is notable, however, that despite most studies concurring that high proliferation is associated with good response to chemotherapy, this same group of patients, as mentioned above, is known to have a poor long-term outcome (Ravaioli *et al*, 1998). A similar type of relationship with outcome is seen with tumour grade. This may reflect a single biological relationship, since mitotic rate is also a component of tumour grade. There was no relationship between pretreatment AI and clinical response, but the GI, which summates the opposing effects of apoptosis and proliferation, did show a relationship with clinical response. The degree of correlation with GI was, however, no greater than that with Ki67 alone. Although biological markers pretreatment predicted clinical response, no relationship was seen with disease-free or overall survival.

The data from this study confirm our earlier findings of a significant increase in apoptosis following chemotherapy and extend them with the observed significant fall in proliferation at 24 h. Since changes in apoptosis and/or proliferation are requisites for changes in tumour growth rate, their early measurement during treatment has been investigated on the basis that this might enable the prediction of eventual clinical response and long-term benefit from treatment. A small pilot study of 15 patients by Chang *et al* (2000) using FNA and different laboratory methods did find a significant relationship between early change in apoptosis (at 24–72 h following treatment) and clinical response (Chang *et al*, 2000). We were unable to confirm such a relationship between changes at 24 h and clinical response.

The timing of sampling post chemotherapy may be important in quantifying the increase in AI: the Chang study sampled at either 24 and/or 72 h postchemotherapy, and used a mean of both measurements when more than one was available. A recent study, again with 15 patients took biopsies at 24 and 48 h, and also failed to show a relationship between apoptosis at 24 h following chemotherapy, but demonstrated a relationship at 48 h. They noted marked variations though, with one tumour showing a rise in apoptosis at 24 h, but not at 48 h (Davis *et al*, 2003). It seems likely that the time of peak increase in apoptosis may vary between patients.

The biopsy at 24 h in our study was taken after starting chemotherapy alone, but tamoxifen was subsequently given to some patients that may have had an effect on tumour response and therefore possibly confounded any relationship between chemotherapy-induced apoptosis and clinical response. We therefore performed an analysis on the data from patients who did not receive tamoxifen, combined with those from patients that were ER negative (where the benefit of any tamoxifen, even if given would have been expected to be negligible). Even in this subgroup no relationship between changes in AI or proliferation at 24 h and clinical response was seen. A recent study, however, has shown no difference in the response rate to primary chemotherapy with the addition of tamoxifen (von Minckwitz *et al*, 2001).

Our study was small, but the number of samples would have enabled a correlation of 0.40 to be detected in 90% of cases. To be clinically useful in predicting response in individual patients, a correlation would need to be substantially greater, and therefore this study would have been able to detect a correlation of clinical utility. Only 62% of patients had enough tissue in the either biopsy to allow assessment of biological markers by immunohistochemistry, principally due to the large numbers of tumour cells needed to evaluate AI with acceptable precision. This introduces a sampling bias, the data being only applicable to the subgroup with repeatedly cellular samples. The Chang study also noted this problem using FNAs, only 15 out of 28 patients (53%) having enough material for biological analysis. A 50–60% success rate also limits the widespread clinical utility of these tests.

Given that there were significant changes in both proliferation and apoptosis after 24 h, which are both likely to contribute to eventual tumour response, we assessed the relationship of GI with clinical response. It was notable that although there were 20 tumours that showed an increase in Ki67 and 10 that showed an decrease in AI, only five showed an increase in the growth index, indicating that the therapy had a beneficial overall effect on tumour growth in 90% of tumours. While the GI pretreatment correlated with response, changes in GI 24 h after chemotherapy had no predictive value for response. Thus, while this index may be useful as a guide to overall changes in tumour dynamics, it does not appear to have utility for response prediction.

One of the most significant predictors of relapse-free survival following primary chemotherapy has been shown to be a pCR (Fisher *et al*, 1998). In our study not all the patients proceeded to surgery, and most of these were patients who achieved a cCR. This confounded the valid assessment of biomarker analysis in relation to pCR.

The lack of a correlation of these early markers with response to chemotherapy or with clinical outcome is disappointing in view of the clear biological rationale for such a relationship and possible reasons for this need to be considered. It should also be noted that a given reduction in proliferation or increase in cell death might lead to regression of a slowly growing tumour, but only slow the growth of a rapidly growing tumour. Thus it may be over simplistic to expect a close relationship between change in these parameters and response measured clinically. Other measurements of response were not performed in this study. Mammography has been shown to be a poorer than clinical measurement (Florentino *et al*, 2001), but ultrasound maybe more useful and could have allowed measurement of volume changes, which has been used in other studies with primary endocrine treatment (Miller *et al*, 2001; Harper-Wynne *et al*, 2002).

Overall we believe this area merits further evaluation, and the study of noninvasive techniques, such as PET may eventually be applicable very early after starting chemotherapy. Also the use of newer techniques such as DNA microarray may improve the predictability of response by combining the change in expression of larger numbers of genes (Assersohn *et al*, 2002). The analysis growth parameter changes at 24 h seem to have little clinical utility for predicting individual patient response, and measurement at later time points (e.g., 14 out of 21 days) may hold greater promise (Dowsett *et al*, 1999).
